# Expedited retrieval of high-quality Usutu virus genomes *via* Nanopore sequencing with and without target enrichment

**DOI:** 10.3389/fmicb.2022.1044316

**Published:** 2022-11-09

**Authors:** Cora M. Holicki, Felicitas Bergmann, Franziska Stoek, Ansgar Schulz, Martin H. Groschup, Ute Ziegler, Balal Sadeghi

**Affiliations:** Institute of Novel and Emerging Infectious Diseases, Friedrich-Loeffler-Institut, Federal Research Institute for Animal Health, Greifswald-Insel Riems, Germany

**Keywords:** USUV, Nanopore sequencing, MinION, long amplicon, Illumina

## Abstract

Usutu virus (USUV) is a mosquito-borne zoonotic virus and one of the causes of flavivirus encephalitis in birds and occasionally in humans. USUV rapidly disperses in a susceptible host and vector environment, as is the case in South and Central Europe. However, compared to other flaviviruses, USUV has received less research attention and there is therefore limited access to whole-genome sequences and also to in-depth phylogenetic and phylodynamic analyses. To ease future molecular studies, this study compares first- (partial sequencing *via* Sanger), second- (Illumina), and third-generation (MinION Nanopore) sequencing platforms for USUV. With emphasis on MinION Nanopore sequencing, cDNA-direct and target-enrichment (amplicon-based) sequencing approaches were validated in parallel. The study was based on four samples from succumbed birds commonly collected throughout Germany. The samples were isolated from various sample matrices, organs as well as blood cruor, and included three different USUV lineages. We concluded that depending on the focus of a research project, amplicon-based MinION Nanopore sequencing can be an ideal cost- and time-effective alternative to Illumina in producing optimal genome coverage. It can be implemented for an array of lab- or field-based objectives, including among others: phylodynamic studies and the analysis of viral quasispecies.

## Introduction

**Figure 1:**
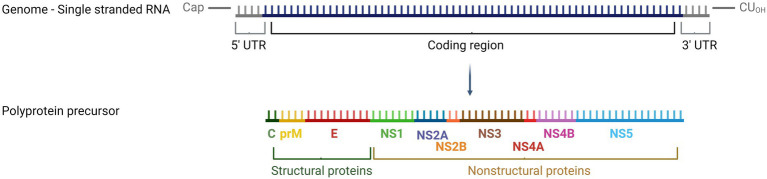
Schematic view of the USUV genome structure. Created with BioRender.com.

Usutu virus (USUV), a neglected Old World flavivirus, causes annually reoccurring epizootics in the avian fauna and sporadic human infections. The enzootic transmission cycle involves ornithophilic mosquitoes (*Culex* spp.) as vectors and birds as reservoir and amplifying hosts. Blackbirds (*Turdus merula*) are particularly susceptible but also other passerine species including song thrushes (*Turdus philomelos*), common kingfishers (*Alcedo atthis*), house sparrows (*Passer domesticus*), canaries (*Serinus canaria forma domestica*), common starlings (*Sturnus vulgaris*), and magpies (*Pica pica*) as well as birds of prey such as owls (*Strigiformes*) can become infected (Becker et al., 2012; Saiz and Blazquez, 2017). USUV is an enveloped virus with a diameter of approximately 40–60 nm. It has a positive sense single-stranded genome of 11,064 nucleotides (nt) harboring a type I cap structure but lacking a poly-A tail (Bakonyi et al., 2004). The genome comprises an open reading frame encoding a single polyprotein (3,434 amino acids) that is cleaved by viral and cellular proteases into three structural (capsid C, premembrane prM, and envelope E) and seven nonstructural proteins (NS1, NS2A, NS2B, NS3, NS4A, NS4B, and NS5) (Figure 1) (Calisher and Gould, 2003).

With its origin in South Africa (Williams et al., 1964), USUV reached Europe at the turn of the 21^st^ century and was detected in Italy in 1996 retrospectively (Weissenböck et al., 2013) and in Austria in 2001 (Weissenböck et al., 2002). From there onwards, it rapidly spread to neighboring countries including Hungary, Switzerland, Croatia, Serbia, Slovakia, and the Czech Republic (Bakonyi et al., 2007; Steinmetz et al., 2011; Hubálek et al., 2014; Kemenesi et al., 2018; Čabanová et al., 2019). In 2010, USUV was detected for the first time in the southwest of Germany in *Culex pipiens* mosquitoes from Weinheim, Baden-Württemberg (Jöst et al., 2011). Mass mortality events of Eurasian blackbirds (*Turdus merula*) followed in 2011, 2016, and 2018 (Becker et al., 2012; Ziegler et al., 2016; Cadar et al., 2017; Lühken et al., 2017; Michel et al., 2019; Zecchin et al., 2021). Concurrently, the geographic range of USUV expanded from the southwest towards the east (Saxony and Saxony-Anhalt) and north (Lower Saxony, Bremen, and Schleswig-Holstein) until in 2018, all national federal states were afflicted (Sieg et al., 2017; Michel et al., 2018, 2019).

USUV isolates cluster into eight distinct lineages (Africa 1, 2, and 3 and Europe 1, 2, 3, 4, and 5), of which many cocirculate throughout Europe (Nikolay, 2015; Engel et al., 2016; Bakonyi et al., 2017; Cadar et al., 2017; Calzolari et al., 2017; Hönig et al., 2019; Zecchin et al., 2021). Until now, five of these lineages have been recorded in Germany: Africa 2, 3, Europe 2, 3, and 5 (Cadar et al., 2015, 2017; Ziegler et al., 2016; Michel et al., 2019). Three are still regularly isolated to date: USUV lineages Europe 3 and Africa 3 are spread throughout Germany (Ziegler et al., 2022), while lineage Europe 2 is only present in the east (Michel et al., 2019; Santos et al., 2021; Ziegler et al., 2022). By contrast, USUV Africa 2 was only found in Germany in the past: in Berlin (2015, 2017, 2018), in Leipzig (2017, 2018), and in Hannover (2018) (Ziegler et al., 2016; Michel et al., 2019; Santos et al., 2021; Störk et al., 2021). USUV Europe 5 was proposed as a novel putative lineage in Bonn in 2014 (Cadar et al., 2015) yet was never isolated again in subsequent years.

Even though partial USUV sequences are available from 2017 to 2020 (Michel et al., 2019; Ziegler et al., 2022), whole-genome sequences of USUV isolates from birds in Germany are limited (Becker et al., 2012; Ziegler et al., 2016; Störk et al., 2021). This makes it difficult to assess geographic and temporal dynamics of USUV in Germany. Whole-genome sequencing (WGS) can also be used to track cases or clusters in outbreak investigations. To ease the implementation of future phylogeographic and phylodynamic USUV studies, we aimed to compare possible sequencing platforms for USUV. In this study, we are comparing traditional low-throughput Sanger sequencing with innovative next-generation (second- and third-generation (SGS and TGS)) high-throughput sequencing technologies. Existing whole-genome sequences in the past were generated using SGS based on Illumina (Illumina, San Diego, CA, United States of America) (Ziegler et al., 2016) or Roche 454 Genome Sequencer FLX system instrument (Roche, Mannheim, Germany) (Becker et al., 2012). In this study, we therefore used TGS (long-read), amplicon and direct DNA sequencing *via* MinION by oxford Nanopore-technology (ONT, Oxford Science Park, the United Kingdom), in order to compare the previously used SGS (short-read), a reversible dye terminator technology *via* Illumina, to that of TGS.

## Materials and methods

### Samples

Liver of a Eurasian blackbird (sample 1), blood coagulum of a great grey owl (*Strix nebulosa*, sample 2), kidney of another great grey owl (sample 3), and cell-culture supernatant (brain) of a steamer duck (*Tachyeres* sp., sample 4), which were found dead in Germany in 2019, were submitted to the national reference laboratory at the Friedrich-Loeffler-Institut (FLI) (Table 1). Viral RNA of samples 1–3 was extracted using the RNeasy Mini Kit (Qiagen, Hilden, Germany) according to the manufacturer’s protocol. RNA from cell-culture supernatant (sample 4) was isolated using the QIAamp Viral RNA Mini Kit (Qiagen) following the manufacturer’s instructions. We selected samples that covered the three most common lineages in Germany (Europe 2, Europe 3, and Africa 3), that were collected from different federal states, and where the RNA was of good quality with high genome copy numbers per μl. USUV infection was detected in all four birds using a published real-time quantitative polymerase chain reaction (RT-qPCR) assay (Jöst et al., 2011) with cycle threshold (Ct) values between 12.22 and 15.11, corresponding to genome copy numbers of 8.31 × 10^8^ and 1.36 × 10^8^ per μl, respectively.

**Table 1:** Sample information.

Sample	ID-number	GenBank number (partial sequence)^*^	GenBank number (WGS)	Order	Common name	Scientific name	Organ	Ct value	Genome copies/μl total RNA	USUV lineage	Origin (city/ federal state)
1	ED-I-74/19	MZ754011	OP422563	*Passeriformes*	Eurasian blackbird	*Turdus merula*	Liver	14.17	6.25 × 10^8^	Europa 3	Wunstorf (LS)
2	ED-I-171/19	MZ754006	OP422564	*Strigiformes*	Great grey owl	*Strix nebulosa*	Blood coagulum	12.22	8.31 × 10^8^	Europa 2	Cottbus (BB)
3	ED-I-44/20	MZ754007	OP422562	*Strigiformes*	Great grey owl	*Strix nebulosa*	Kidney	13.68	3.58 × 10^8^	Africa 3	Kirchham (BY)
4	19 51217-UGF187	MZ779076	OP422565	*Anseriformes*	Steamer duck	*Tachyeres* sp.	Brain^†^	15.11	1.36 × 10^8^	Africa 3	Berlin (BE)

LS, Lower Saxony; BB, Brandenburg; BY, Bavaria; BE, Berlin.

* Partially sequenced in (Ziegler et al., 2022).

† Cell-culture supernatant on Vero cells.

## Nanopore sequencing

### Sample preparation

#### Direct DNA sequencing (rapid barcoding)

For direct cDNA sequencing, synthesis of complementary DNA (cDNA) was performed *via* the multiplex PCR as previously described by Quick et al., 2017 using the SuperScript IV First-Strand Synthesis System (Cat. no. 18091050; Invitrogen by Thermo Fisher Scientific, Darmstadt, Germany) and random primers (Invitrogen) for reverse transcription (Table 2). After cDNA synthesis, the Rapid Barcoding Sequencing Kit (SQK-RBK004; ONT) and the Flow Cell Priming Kit (EXP-FLP002; ONT) were used according to the manufacturer’s instructions after a purification step with the Agencourt AMPure XP beads (Agencourt, Beckman-Coulter, United States) (Table 2).

**Table 2:** List of kits needed for direct DNA sequencing (MinION direct) and amplicon sequencing (MinION amplicon).

Kits	MinION direct	MinION amplicon
SuperScript IV First-Strand cDNA Synthesis Reaction Kit (Invitrogen)	X	X
Agencourt AMPure XP beads (Agencourt)	X	X
Rapid Barcoding Sequencing Kit SQK-RBK004 (ONT)	X	
Flow Cell Priming Kit EXP-FLP002 (ONT)	X	X
1 D Native barcoding genomic DNA Kit EXP-NBD104 (ONT)		X
Ligation Sequencing Kit SQK-LSK109 (ONT)		X
NEBNext Ultra II End Repair/dA-Tailing Module (New England Biolabs)		X
NEBNext Ultra II Ligation Module (New England Biolabs)		X
NEBNext Quick Ligation Module (New England Biolabs)		X

#### Amplicon sequencing (native barcoding)

For amplicon sequencing, a conventional whole-genome PCR for USUV was performed as previously described (Oude Munnink et al., 2019). For this purpose, 32 primer pairs were used in two different reactions that generate overlapping amplicons with a length of 500 base pairs (bp) using the AccuPrime Taq DNA Polymerase High Fidelity (Cat. no. 12346-086; Invitrogen). For the quantification of the purified PCR products, NanoDrop (NanoDrop 2000c Spectrophotometer; Thermo Fisher Scientific) measurement was performed. The presence of amplicons of the correct length was determined by gel electrophoresis (1.5% agarose gel). To remove possible chemical contaminations, which could affect library preparation efficiency and sequencing quality, a purification step with Agencourt AMPure XP beads (Agencourt) was performed. To prepare the end-prep mix and barcoding/ligation master mixes, NEBNext Ultra II End Repair/dA-Tailing Module, NEBNext Ultra II Ligation Module, and NEBNext Quick Ligation Module (New England Biolabs, Ipswich, United States) were used in a separate hood for the preparation of master mixes to avoid cross-contaminations. Using the 1 D Native barcoding genomic DNA Kit (with EXP-NBD104 and SQK-LSK109; ONT) and the Flow Cell Priming Kit (EXP-FLP002; ONT) MinION sequencing was performed according to the manufacturer’s instructions. Spot-ON flow cells (R9.4.1; ONT) were used in a MinION MK1c instrument (ONT) for both approaches. Typically, 8 h of sequencing was sufficient to generate enough data for 6 samples multiplexed on one flow cell.

### Illumina sequencing

Viral nucleic acid was directly sent on dry ice to Eurofins Genomics Europe Sequencing GmbH, Ebersberg, Germany, and was sequenced *via* a special service for RNA-virus Sequencing (INVIEW virus sequencing, Eurofins Genomics Europe Sequencing GmbH). Sequencing was performed using proprietary methods of Eurofins Genomics Europe Sequencing GmbH and the Illumina NovaSeq 6,000 platform (2 × 150 Sequence mode). Library preparation was performed with the NEBNext Ultra II Directional RNA Library Prep Kit for Illumina (New England Biolabs) and quality measurement of the mRNA was completed using the Fragment analyzer (ABI DNA analyzer; Applied Biosystems by Thermo Fischer Scientific).

### Sanger sequencing

For Sanger sequencing, USUV-specific oligonucleotide primers (3 primer pairs) were chosen to amplify a partial segment of the envelope-coding gene (length of 1,066 nt), according to Eiden et al. (2018) and Ziegler et al. (2022). For the amplification, a SuperScript III One-Step RT-PCR System with Platinum Taq DNA Polymerase (Invitrogen) and the multi-block PCR thermal cycler Biometra TRIO (Analytik Jena GmbH, Jena, Germany) were used. A purification was performed using gel electrophoresis (1.5% agarose gel). For visualization with blue light, the gel was stained with the SYBR Safe DNA gel stain (Invitrogen). DNA bands were cut out and purified with the Wizard SV Gel and PCR Clean-Up System (Promega, Walldorf, Germany). Samples were sequenced *via* the TubeSeq service of Eurofins (Eurofins Genomics Europe Sequencing GmbH). The Sanger partial sequences were already published in Ziegler et al., 2022, yet for comparison purposes, various sequencing methods and results were included in this study.

### Data analysis for Illumina sequencing

Raw read quality was assessed using FastQC (Sena Brandine and Smith, 2019) and residual adapter sequences were trimmed using Cutadapt v4.1 (Martin, 2011). Trimmed reads were mapped against the USUV reference genome (GenBank accession number: NC_006551.1; Moureau et al., 2015) using Bowtie2 v2.4.5 (Langmead and Salzberg, 2012). The amplicon coverage was normalized to 50 using BBNorm (Bushnell, 2014) after which a *de novo* assembly was performed using SPAdes (Bankevich et al., 2012). Raw, quality-controlled reads were mapped back against the obtained consensus genome using Geneious Prime 2021.0.1 (Biomatters Ltd., Auckland, New Zealand).

### Data analysis MinION sequencing

Fast5 raw data reads were demultiplexed using Guppy v4.5.4 (Wick et al., 2019) and Porechop v0.2.4.^1^ Primers were trimmed and reads were quality controlled to a minimal length of 200 bp and a median Phred score of 7 using QUASR (Watson et al., 2013). First, a reference-based alignment against the selected USUV reference genomes v23 (Goodacre et al., 2018) was performed in KMA (k-mer alignment) (Clausen et al., 2018). The consensus genome was extracted and compared to the non-redundant database using Blastn (Altschul et al., 1990). Subsequently, the closest relative sequence was selected and used for a second reference-based alignment using the quality-controlled reads in Minimap2 (Li, 2018). Consensus sequences were visualized with Geneious Prime 2021.0.1 (Biomatters Ltd.).

### Phylogenetic analysis

The Muscle algorithm was used to align the sequences (Edgar, 2004). The best model of nucleotide substitutions (GTR + I + G4) was selected using jModeltest v.2 (Darriba et al., 2012), and maximum likelihood trees were reconstructed using PAUP* v.4 (Swofford, 2003). Reliability of the obtained tree topologies was performed by bootstrap testing (1,000 replicates), and finalized trees were reconstructed with FigTree v.1.4.3 (Rambaut, 2012).

## Results

**Figure 2:**
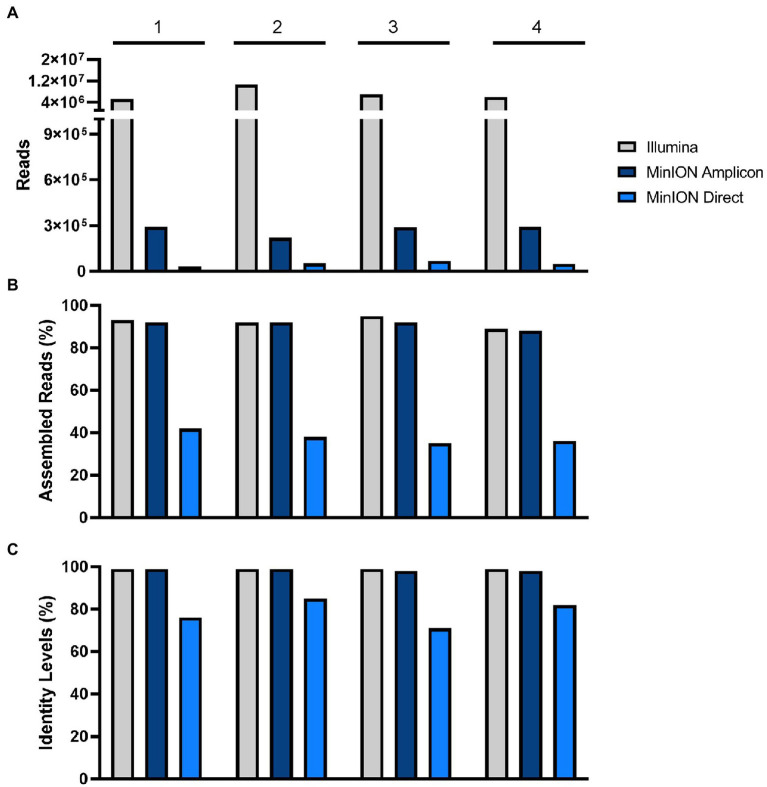
Comparison of **(A)** total read counts, **(B)** assembled reads (%), and **(C)** identity levels (%). in Illumina, MinION amplicon, and MinION direct in samples 1–4.

In this study, we compared the performance of Nanopore and Illumina sequencing platforms, using three different methods: direct DNA sequencing and amplicon sequencing were performed with the MinION device and RNA sequencing was done with Illumina. For MinION direct (Nanopore) and massive parallel sequencing (Illumina), no amplicon-enrichment steps were used. For Illumina, the average and maximum USUV target sequence read lengths were as expected short with 148 and 246 nt, respectively. For MinION direct and amplicon, the USUV target sequence read lengths were, on average 282 nt and 578 nt, respectively, and the maximum lengths were 631 nt and 1,072 nt, respectively. Even though, the average read lengths of the USUV target sequences were therefore longer for MinION amplicon (578 bp vs. 282 bp), this was not the case for the non-target sequences, where MinION direct produced longer read lengths compared to MinION amplicon (≈1,750 bp vs. 500 bp). Total read counts, assembled reads, and identity levels are summarized in Figure 2 for all samples. Coverage, depth, read quality, contig length, and identity levels against USUV reference genome (GenBank accession number: NC_006551.1) are among others summarized in Table 3.

**Table 3:** Comparison of the most important whole-genome sequencing quality parameters in Illumina, MinION amplicon, and MinION direct.

Sample	Coverage (%)	Mean read quality (Phred Quality Score)	Estimated contig length N50 (bp)	Identity levels (%)	Assembled reads (%)	Depth (average log_10_ transformed)	Number of total reads	Total bases	Final error rate (%; substitution and deletion)
**Illumina**									
1	99	35.3	2,742	99	93	4.3	5,268,103	1.6 Gb	0.15
2	99	35.2	2,176	99	92	3.8	10,667,810	4.3 Gb	0.12
3	99	35.5	3,862	99	95	2.9	6,959,105	2.1 Gb	0.11
4	99	33.4	2,432	99	89	3.9	6,030,472	1.9 Gb	0.18
**MinION amplicon**									
1	99	25	3,176	99	92	4.0	292,000	1.2 Gb	2.02
2	99	22	2,872	99	92	3.2	220,000	980 Mb	1.86
3	99	23	4,152	98	92	2.4	288,000	1.1 Gb	1.62
4	99	22	2,766	98	88	3.3	292,000	1.2 Gb	2.28
**MinION direct**									
1	52	19	800	76	42	1.0	32,000	80 Mb	≥4
2	64	18	755	85	38	0.8	52,000	122 Mb	≥4
3	75	20	900	71	35	0.5	68,000	151 Mb	≥4
4	49	20	920	82	36	0.8	48,000	98 Mb	≥4

Gb, Gigabyte; Mb, Megabyte.

**Figure 3:**
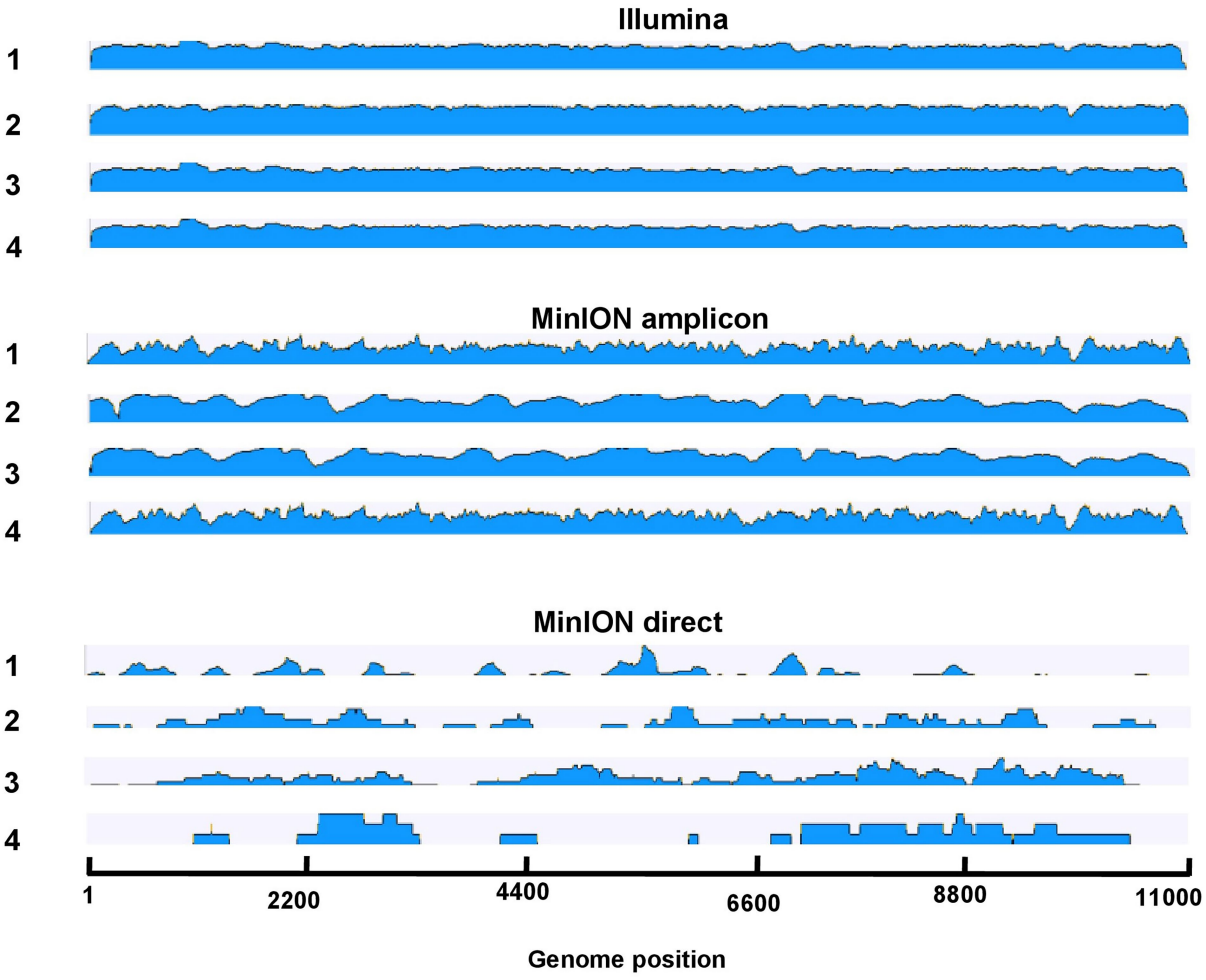
Overview of read coverage of sequenced genomes in Illumina, MinION amplicon, and MinION direct in samples 1–4. The scale represents the log transformed coverage depth (min: 10^1^ and max: 10^6^).

MinION amplicon sequencing and RNA sequencing with Illumina had a higher efficiency in comparison to the results from the MinION direct DNA sequencing protocol. The data of the MinION amplicon and Illumina sequencing indicate that more specific reads can be obtained with these methods (Table 3; Figure 2). Nonetheless, a minimal depth (10^0.5^–10^1^ reads) and genome coverage (49–75%) were obtained using MinION direct DNA sequencing (Table 3; Figure 3). With the exception of direct DNA sequencing, genome assembly was successfully completed for all four samples using various sequencing platforms: MinION amplicon and Illumina (Table 3). Identities of more than 98% with consensus sequences based on the reference strain were achieved for all four genomes (Table 3; Figure 2). We measured the error rates of the various sequencing platforms by aligning the generated reads to the appropriate reference sequences. Subsequently, the nanopore assemblies were compared to the data generated by Illumina, which was used to assemble the USUV virus genomes. The error rate metrics from Illumina sequencing were better than those from MinION amplicon. The error rates were estimated at 0.15, 0.12, 0.11, and 0.18% for Illumina and 2.02, 1.86, 1.62, and 2.28% for MinION amplicon for samples 1–4, respectively.

**Figure 4:**
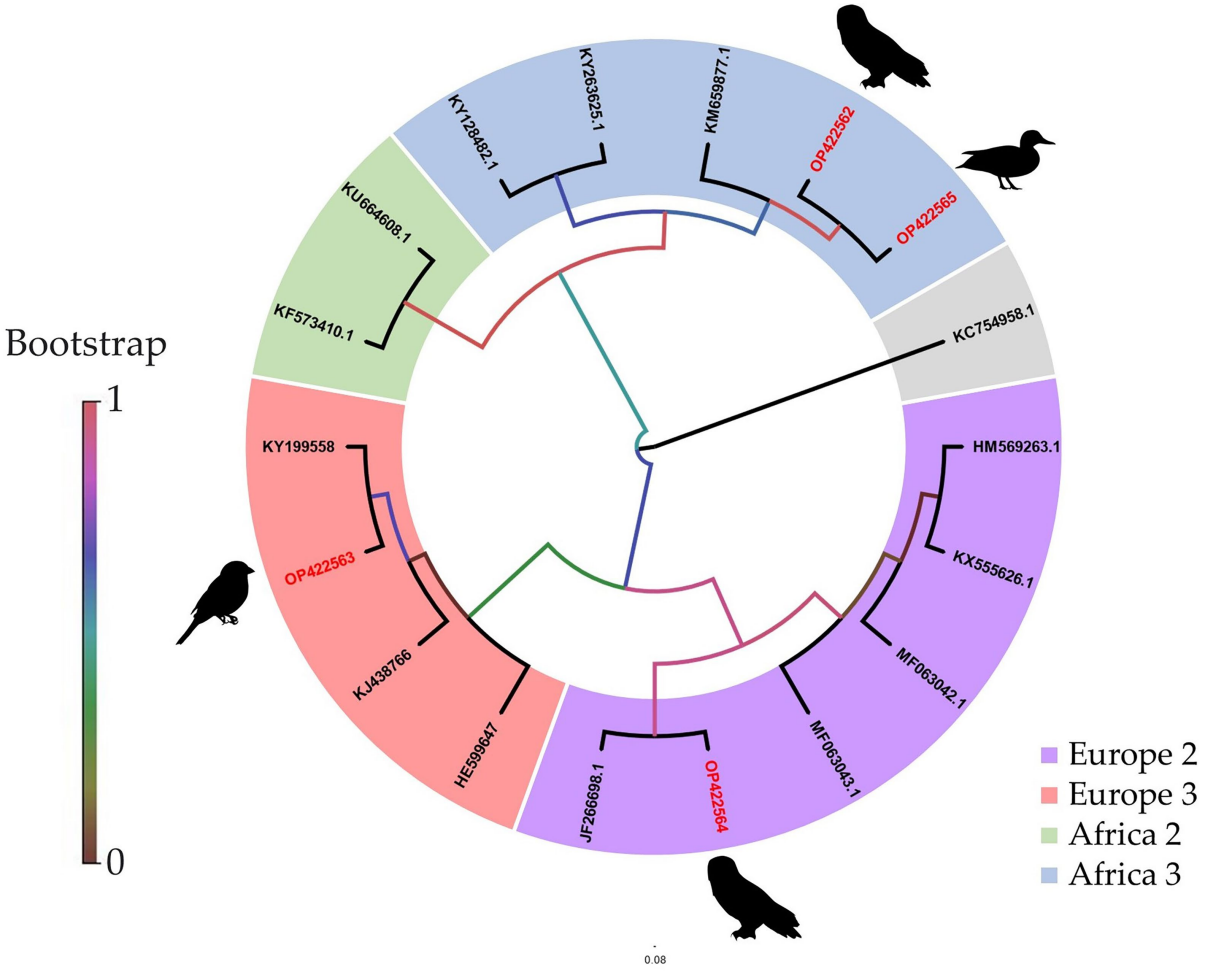
Phylogenetic analysis of USUV whole-genome strains detected in birds from Germany. The sequences from Germany generated in this study *via* MinION amplicon are highlighted in red. Sequences are labeled by codes containing the GenBank accession number. The phylogenetic trees were identical independent of the platform used (Sanger, Illumina, or MinION amplicon) and therefore only one is illustrated here.

In comparison to the other two sequencing approaches tested, only an incomplete genome coverage including many gaps in the sequences was obtained from MinION direct DNA sequencing. The best quality and quantity of sequencing results were achieved by Illumina sequencing (Table 3). For each sample, Illumina produced on average more reads (total reads: ≈7.2 million reads; USUV reads: ≈6.2 million reads) with more depth (10^3.7^ reads). The percentage of USUV target reads generated with the Illumina platform for the four samples (1–4) was 79, 91, 87, and 85%, respectively. On the other hand, MinION direct DNA sequencing turned out to be the cheapest alternative compared to other SGS and TGS protocols (Tables 2 and 4). As commonly implemented in virus diagnostics, partial sequencing in this study sufficed in identifying the correct USUV lineage of a sample (Ziegler et al., 2022). This can be derived from the three identical phylogenetic trees generated from the various sequencing techniques (one shown as exemplary in Figure 4).

**Table 4:** Comparison of the most important genome sequencing variables in Sanger (partial genome sequencing), and Illumina, MinION amplicon, and MinION direct (whole-genome sequencing).

Sequence method	Partial sequencing	Whole-genome sequencing		
	Sanger (Chain termination method)	Massive parallel sequencing	MinION amplicon	MinION direct
Platform	Applied Biosystems 3,500 XL Genetic Analyzer	Illumina (Illumina NovaSeq 6,000)	Nanopore (Mk1C)	Nanopore (Mk1C)
Costs of the instrument^*^	€143,600	€850,000	€4,410	€4,410
Costs per sample^*^	≈€40	≈€300	≈€150	≈€100
Time wetlab^*^	27 h	12 d^†^	18 h	14 h
Time drylab^*^	1 h	8 h	6 h	4 h
Space requirement	+++	+++	+	+
Virus-specific PCR required	Yes	No	Yes	No
Portable sequencer	No	No	Yes	Yes

+ = low, ++ = medium, +++ = high.

* Estimated time and costs are laboratory dependent.

† As stated by Eurofins Genomics Europe Sequencing GmbH, 2022.

**Figure 5:**
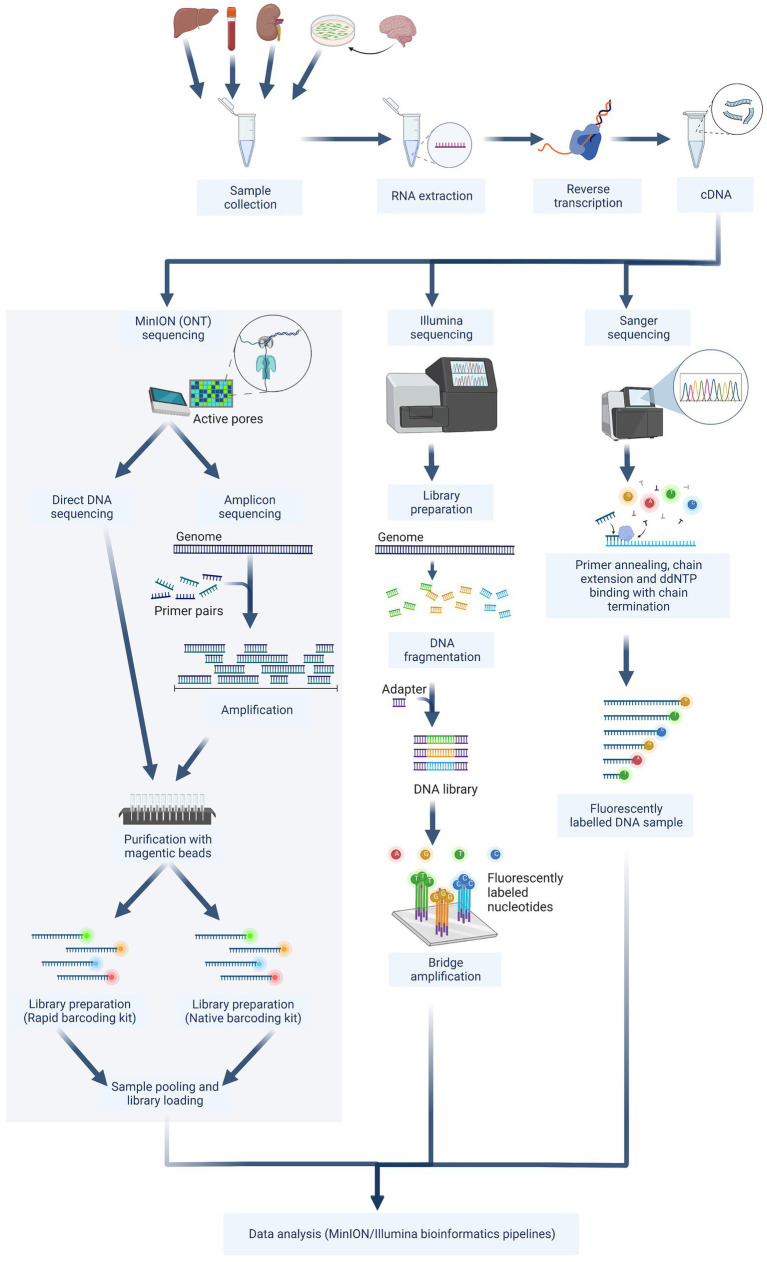
The next-generation sequencing workflow contains three sequencing approaches. The schematic figure was drawn with Biorender.com. ddNTP, Dideoxyadenosine triphosphate.

In recent years, WGS has become a reliable alternative and complement to “traditional” sequencing techniques. As a result, we assessed the efficiency of WGS benchtop equipment as a tool for real-time genomics in rapid-response diagnostics. Different sequencing approaches were also evaluated based on the time to result, the costs of the sequencing instrument, the costs per sample sequenced, the specificity, and the suitability for sensitive WGS (Table 4). The MinION direct DNA sequencing results demonstrated that the entire genome could not be covered using this method. Therefore, comparison to Illumina and amplicon MinION sequencing is pointless. Illumina and amplicon MinION sequencing require the same benchtop laboratory tools for library preparation (Figure 5), although the MinION is more efficient and versatile in terms of run time (Table 4). It was possible to achieve a sequence consensus accuracy of 90% within a few hours of the sequencing run, as opposed to an incompressible run time of 20 h for the Illumina system. While the Illumina system is stand-alone, at least during the initial stages of bioinformatics, such as base calling and demultiplexing, MinION requires a strong laptop computer. Even though ONT has recently increased the sequencing accuracy of the MinION (currently, Nanoporetech has developed Bonito, A PyTorch Basecaller for Oxford Nanopore Reads with higher accuracy), raw read error rates remain higher than those of Illumina (around 0.2%), requiring a greater read depth to produce a reliable consensus sequence.

## Discussion

To date, USUV epidemiological surveillance programs have focused primarily on the molecular (RT-qPCR) and serological detection (virus neutralization tests and enzyme-linked immunosorbent assays) of USUV-specific genome and antibodies, respectively. Passive and active surveillance efforts of wild birds and captive zoological birds are regularly performed, among others in Austria (Bakonyi et al., 2007; Chvala et al., 2007; Meister et al., 2008; Rubel et al., 2008), Belgium (Rouffaer et al., 2018; Benzarti et al., 2020), France (Vittecoq et al., 2013; Roesch et al., 2019; Constant et al., 2020), Germany (Ziegler et al., 2015, 2022; Michel et al., 2018, 2019), Hungary (Bakonyi et al., 2007; Weidinger et al., 2020), Italy (Manarolla et al., 2010; Savini et al., 2011; Tamba et al., 2011; Giglia et al., 2021; Lauriano et al., 2021; Scaramozzino et al., 2021; Zecchin et al., 2021; Mancuso et al., 2022), the Netherlands (Lim et al., 2018), Spain (Jurado-Tarifa et al., 2016; Marzal et al., 2022), and the United Kingdom (Buckley et al., 2003; Horton et al., 2013; Folly et al., 2020). Nonetheless, a genomic surveillance of USUV is rare, with a limited number of available full-genome sequences. The majority of these derived from initial USUV detections in European countries and resultant surveillance efforts, including Italy (Savini et al., 2011; Calzolari et al., 2013; Gaibani et al., 2013; Zecchin et al., 2021), Germany (Jöst et al., 2011; Becker et al., 2012; Cadar et al., 2017; Santos et al., 2021), and the Netherlands (Cadar et al., 2017; Oude Munnink et al., 2020b). Individual sequences were isolated in Austria (Bakonyi et al., 2004, 2017; Chvala et al., 2007; Weidinger et al., 2020), Belgium (Garigliany et al., 2014, 2017; Benzarti et al., 2020), France (Cadar et al., 2017), Hungary (Bakonyi et al., 2007), and Spain (Vázquez et al., 2011; Höfle et al., 2013; Bakonyi et al., 2014). Outside of Europe, USUV has been fully sequenced from human patients, rodents, and vectors (*Culex* spp.) from the Central African Republic and Senegal, Africa (Bakonyi et al., 2004; Nikolay et al., 2013; Diagne et al., 2019). Similarly, reference strains are accessible from mosquitoes from Israel (*Culex* spp. and *Aedes albopictus*; Mannasse et al., 2017). The aim of this study was to test MinION Nanopore sequencing, promoted as a cost- and time-effective sequencing technique, for its user-friendly application in ongoing phylogenetic analyses and to compare the results to those obtained by first- and second-generation sequencing platforms.

### First-generation sequencing vs. next-generation sequencing

NGS techniques, including MinION Nanopore and Illumina platforms, allow the simultaneous deep sequencing of millions of base pairs from multiple samples, while Sanger et al. (1977) can only sequence one DNA fragment at a time. Sanger is still the “gold-standard” in screening virus variants (i.e., sequencing single amplicon targets), especially when screening low sample numbers. As described in a previous study (Ziegler et al., 2022), Sanger sequencing consistently unraveled the USUV lineage affiliations of the tested samples. By contrast, NGS whole-genome sequencing platforms are ideal when a higher target sequence size is required. They can generate a more comprehensive sequencing dataset with greater depth and are, therefore, more sensitive in detecting low-frequency variants (<1%) as well as discovering novel gene variants in combination with a higher number of low-abundance samples (low input quantity and quality; Shendure et al., 2017; Young and Gillung, 2020). Through mass parallelization of sequencing reactions, NGS decreased the necessary costs and turnaround time per sample and revolutionized genomics (Margulies et al., 2005). It opened the door to an array of approaches including viral evolution, metagenomics, and transcriptomics in the study of quasispecies (Radford et al., 2012; Quer et al., 2022). These are genetically closely related viruses that can exist concurrently in a susceptible vector or host due to the high mutation rate of RNA viruses. This has been described for numerous flaviviruses, such as Zika virus (van Boheemen et al., 2017) and West Nile virus (Dridi et al., 2015). Understanding the generation and composition of these quasispecies can give insights into virus adaptation and immune escape mechanisms.

### Second-generation sequencing vs. third-generation sequencing

TGS stands for single-molecule sequencing as well as real-time sequencing with the possibility of omitting the requirement for DNA amplification (Heather and Chain, 2016). When directly comparing SGS, *via* Illumina or Ion Torrent (Thermo Fisher Scientific), with TGS, *via* for example SMRT (Single molecule real time; Pacific Biosciences, United States) or MinION (ONT), the main difference is the length of the reads. An advantage of SGS is the high throughput with fairly low error rates (≈1–2.4%) compared to TGS (≈10–15%). The disadvantage is linked to the shorter read lengths (75–600 nt/read) compared to TGS (60 K–2 M bp), which can limit the detection of mutations (deletions or insertions) as well as the characterization of repetitive genomic regions (Quer et al., 2022). In this study, however, both the SGS (Illumina) and the TGS techniques *via* amplicons (MinION; excluding direct sequencing) produced similar results with high identity levels of 98–99%. Not surprisingly, the only difference being that Illumina produced significantly more reads than MinION with an average of 7,231,373 compared to 273,000 reads, respectively. This difference in read counts and consequently also in depth could be relevant when evaluating mutation sites, primarily those in highly variable regions. It can be concluded that both platforms are equally suitable for genotype classification. However, it should not be disregarded that due to the novelty of Nanopore sequencing with its quick turnaround, there is a flood of continuously tailored flow cells, library preparation kits, protocols, and analytical software on the market, making a standardization of results as for Illumina more difficult (Stefan et al., 2022).

### Third-generation sequencing: Amplicon-based vs. direct DNA sequencing

For the Nanopore platform *via* MinION an amplicon-based sequencing technique was used as well as a newly developed direct DNA sequencing method. Studies directly comparing these two strategies are still scarce, even though both of their applications were tested for example for Porcine Reproductive and Respiratory Syndrome Virus (PPRSV) (Tan et al., 2019) and the mycovirus, CHV-1 (Leigh et al., 2020). Amplicon-based sequencing using specific USUV primers has the advantage that lower viral titers can be successfully sequenced than in a non-selective approach including direct/SISPA (Sequence-independent, single-primer amplification) sequencing (Reyes and Kim, 1991). One of the limitations, however, is that the overlapping primer pairs need to be regularly revised to cover all available USUV sequences to try and reduce the possibility of missing out on certain genomic variations (Cotten et al., 2014; Cadar et al., 2017; Oude Munnink et al., 2020a,b). Aside from optimizing variant detection, primer concentrations need to be tailored to allow an even amplicon balance and a good coverage (Gohl et al., 2020). In this study, the primers used had already been published by Oude Munnink et al., 2019 and were mixed into two separated mixes to avoid overlapping fragments. After the analysis of USUV sequences *via* MinION amplicon (Figure 5), results indicated that the coverage of specific genome positions needs to be improved (for example positions between 1 and 1,000 bp, 1,800 and 2,500 bp, and 9,500 and 10,500 bp). Accordingly, the used primers should still be fine-tuned for future studies, as certain primer pairs produced more coverage than others (Santos et al., 2021).

An alternative to amplicon-based sequencing would be a random amplification approach *via* direct DNA sequencing kits or SISPA, as often used for metagenomic studies. These methods have an even shorter turnaround time, an easily deployable protocol, and lack systematic sequencing bias, therefore reducing the need for regular reappraisal of the protocol. Metagenomics allow the detection of new as well as known highly diverse viral agents without prior clinical knowledge (Kafetzopoulou et al., 2018). In this study, we tested direct sequencing of USUV cDNA but were not able to produce a sufficient dataset. It resulted in a smaller data set than amplicon-based sequencing, with lower read numbers (32,000 compared to 22,000 reads), quality of reads (18 compared to 25 Phred Quality Score), quantity of assembled reads (36% compared to 92%), and identity levels (71% compared to 92%). Due to the very short contig lengths (<1,000 bp) and the numerous gaps in the sequence, a poor coverage was observed and it was not possible to generate a consensus sequence based on the direct approach. Base calling errors observed as substitutions, insertions/deletions (indels), and ambiguous bases were identified in the generated data. These errors were more frequent in samples with lower total or target read counts. Allowing a longer sequencing time of the MinION device did not increase data yield and quality. It appears that the sequencing of cDNA without further target-amplification steps is less efficient at producing the required coverage of the region of interest, thereby requiring a higher DNA input. Consequently, in our study, a success was more guaranteed when enriching the input DNA with PCR amplification using target-specific primers. Skipping prior enrichment- and PCR-steps, this technique (cDNA + MinION rapid barcoding kit) is optimized for speed (>1 d) and simplicity and not for the achievement of high throughput. Such untargeted methods are not optimal for large-scale surveillance programs with varying sample quality as they have a lower detection limit requiring higher clinical titers (Faria et al., 2017; Quick et al., 2017). Therefore, in this study, direct sequencing appeared to be more error-prone, and for future phylogenetic studies with known pathogens like USUV, focus on targeted amplicon-based sequencing efforts is recommended.

### Possible improvements

Future improvement strategies aside from refining the primer pairs/mixes could include testing which multiplexing strategy produces the best data yield and data evenness and whether the incorporation of a washing step of the flow cell can enable the reuse of flow cells, to reduce the sequencing cost per sample. Furthermore, no difference in quality could be observed between the full-genome from cell-culture supernatant and that from organ samples, making an enrichment *via* culture isolation unnecessary in the presence of high viral loads (Houldcroft et al., 2017). Future studies can, therefore, use a broad panel of sample types (e.g., RNA from blood and tissue samples). Similarly, they can also include samples with higher Ct values (≥30) in the diagnostic RT-qPCR. As already described by other studies, it could also be tested whether the prior Agencourt AMPure XP bead (Agencourt) clean-up step of the unbarcoded amplicons could be omitted (Freed and Silander, 2021).

## Conclusion

In this work, we compare four sequencing approaches using USUV and discuss their performance in terms of sensitivity and WGS comprehensiveness. In conclusion, Nanopore technologies including MinION amplicon have boosted WGS on a global scale. Due to the simplicity and portability of the sequencer, sequencing can now be deployed outside of core services, as in the field, as shown for Ebola (Hayden, 2015). Furthermore, data can be obtained at a much shorter turnaround (real-time sequencing) allowing a quick and robust analysis of samples during epidemics or ongoing surveillance efforts. Even though the yield quality is not as high as after employing SGS, it suffices to monitor the emergence and spread of viruses in the frame of phylogeographic studies. Keeping in mind that the platform best suited for a project not only depends on the research question to be answered but also on the scale of the project, the allocated time frame, the available budget, and the available amenities and know-how. Even a combination of approaches may be essential to acquire the most accurate genome assemblies of novel pathogens or highly rare variants (Neal-McKinney et al., 2021). This work provides a deep insight into the characteristics of each method and helps to ease decision-making.

## Data availability statement

The datasets presented in this study can be found in online repositories. The names of the repository/repositories and accession number(s) can be found at: https://www.ncbi.nlm.nih.gov/genbank/, OP422563; https://www.ncbi.nlm.nih.gov/genbank/, OP422564; https://www.ncbi.nlm.nih.gov/genbank/, OP422562; https://www.ncbi.nlm.nih.gov/genbank/, OP422565.

## Author contributions

CH, FB, BS, MG, and UZ: conceptualization. CH, FB, FS, AS, and BS: methodology. CH, FB, and BS: data curation, software, formal analysis, writing—original draft preparation, and visualization. CH, FB, BS, FS, AS, UZ, and MG: validation and writing—review and editing. CH and FB: investigation. UZ and MG: resources, project administration, and funding acquisition. BS, UZ, and MG: supervision. All authors contributed to the article and approved the submitted version.

## Funding

This work was financially supported by the German Federal Ministry of Food and Agriculture (BMEL) through the Federal Office for Agriculture and Food (BLE grant number 2819113919), by the German Ministry of Education and Research (BMBF grant number 01KI2026D) as well as in the frame of the German Centre for Infection Research (BMBF-DZIF grant TTU EI 01.804_00).

## Conflict of interest

The authors declare that the research was conducted in the absence of any commercial or financial relationships that could be construed as a potential conflict of interest.

## Publisher’s note

All claims expressed in this article are solely those of the authors and do not necessarily represent those of their affiliated organizations, or those of the publisher, the editors and the reviewers. Any product that may be evaluated in this article, or claim that may be made by its manufacturer, is not guaranteed or endorsed by the publisher.

## Footnotes

^1^ https://github.com/rrwick/Porechop
